# Supplementary material for: The Evolution and Characterization of the RNA Interference Pathways in Lophotrochozoa
Source: Genome Biol Evol. 2024 May 7;16(5):evae098. doi: 10.1093/gbe/evae098 (PMC11114477; doi:10.1093/gbe/evae098)
Supplement: evae098_Supplementary_Data [file evae098_supplementary_data.zip › Supplementary_materials.zip!/selected_miRNAs/NoGe_selected_miRNAs/mirdeep_pdf_files/NMRB01001254.1_Notospermus_geniculatus_strain_Ushimado_scaffold1254,_whole_genome_shotgun_sequence_26698.pdf]

Provisional ID : NMRB01001254.1_Notospermus_geniculatus_strain_Ushimado_scaffold1254,_whole_genome_shotgun_sequence_26698
Score total : 4699.1
Score for star read(s) : 3.9
Score for read counts : 4693.6
Score for mfe : -1.3
Score for randfold : 0
Score for cons. seed : 3
Total read count : 9218
Mature read count : 9110
Loop read count : 0
Star read count : 108

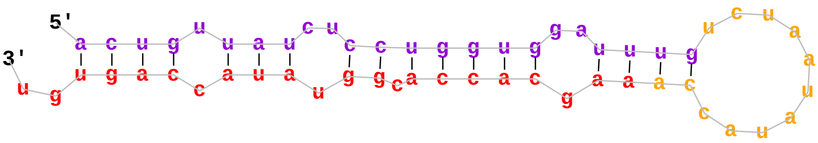

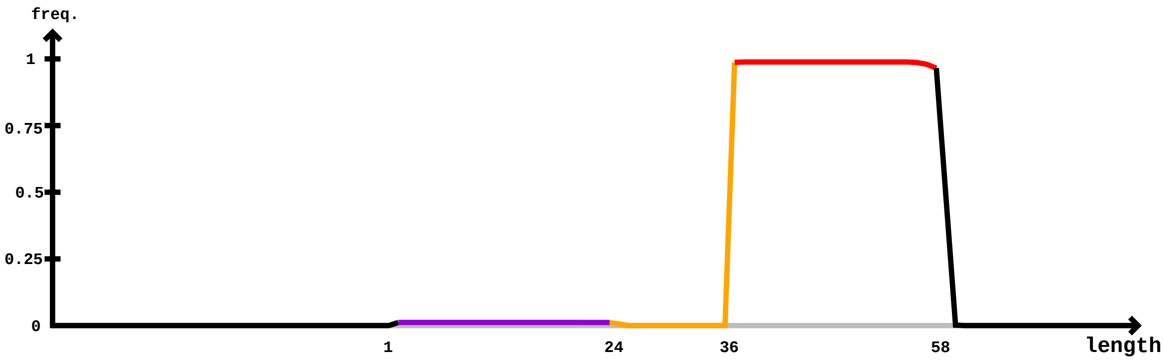


```
                                  Star                                 Mature
5'- aacuugacaaugacuucaaucucaaggcacuugcacuguuaucuccugguggauuugucuaauauaccaaagcaccacgguauaccagugugaauugucugaauuccuacg   -3'    obs
    aacuugacaaugacuucaaucucaaggcacuugcacuguuaucuccugguggauuugucuaauauaccaaagcaccacgguauaccagugugaauugucugaauuccuacg          exp
    ...((((........))))..(((.((((.((((((((.(((..(((((((..((((.........)))).))))).)).))).))))))))..)))))))..........    reads    mm    sample
    ..................................acuguuaucuccugguggauuu.......................................................    1        0     Fm3
    ..................................acuguuaucuccuggugCauuug......................................................    1        1     Fm3
    ..................................acuguuaucuccugguggauuug......................................................    17       0     Fm3
    ..................................acuguuaucuccugguCgauuug......................................................    1        1     Fm3
    ....................................................................aagcaccacgguauacAag........................    1        1     Fm3
    ....................................................................aagcaccacgguauaccag........................    18       0     Fm3
    ....................................................................aagcaccacgguauaccagu.......................    21       0     Fm3
    ....................................................................aagcaccacAguauaccagu.......................    6        1     Fm3
    ....................................................................aagcaccacgguauaccCgug......................    1        1     Fm3
    ....................................................................aagcaccacgguauaccagug......................    60       0     Fm3
    ....................................................................aagcaccacAguauaccagugu.....................    1        1     Fm3
    ....................................................................aagcaccacgguauaUcagugu.....................    1        1     Fm3
    ....................................................................aagcaccacgguauacUagugu.....................    6        1     Fm3
    ....................................................................aagcaccacgguauaccagugu.....................    1612     0     Fm3
    ....................................................................Nagcaccacgguauaccagugu.....................    3        1     Fm3
    ....................................................................aagcaccacgguauaccagGgu.....................    1        1     Fm3
    ....................................................................aagcaccacgguauaccCgugu.....................    5        1     Fm3
    ....................................................................aagcaccacgguauaccGgugu.....................    6        1     Fm3
    ....................................................................aagcaccacgAuauaccagugu.....................    1        1     Fm3
    ....................................................................aagcacNacgguauaccagugu.....................    1        1     Fm3
    ....................................................................aagcaccacgguauaccUgugu.....................    4        1     Fm3
    ....................................................................aaUcaccacgguauaccagugu.....................    3        1     Fm3
    ....................................................................aagcacUacgguauaccagugu.....................    1        1     Fm3
    ....................................................................aagcaccUcgguauaccagugu.....................    1        1     Fm3
    ....................................................................aagcaccacgguauacGagugu.....................    1        1     Fm3
    ....................................................................aagcaccacgUuauaccagugu.....................    2        1     Fm3
    ....................................................................aagcaccacgguauaGcagugu.....................    2        1     Fm3
    ....................................................................aagcaccacgguauacAagugu.....................    7        1     Fm3
    ....................................................................Uagcaccacgguauaccagugu.....................    2        1     Fm3
    ....................................................................aagUaccacgguauaccagugu.....................    2        1     Fm3
    ....................................................................aagcaccacgguauaccaguguU....................    8        1     Fm3
    .....................................................................agcaccacgguauaccagugu.....................    3        0     Fm3

    ..................................acuguuaucuccugguggauuug......................................................    8        0     Mm3
```

```
                                  Star                              Mature
aacuugacaaugacuucaaucucaaggcacuugcacuguuaucuccugguggauuugucuaauauaccaaagcaccacgguauaccagugugaauugucugaauuccuacg
..................................acuguuaucuccuggugCauuug......................................................   1      1     Mm3
.....................................................................aagcaccacgguauaccagu......................   7      0     Mm3
.....................................................................aagcaccacgguauaccagug.....................   5      0     Mm3
.....................................................................aagcaccacgguauaccagugG....................   11     1     Mm3
.....................................................................aagcaccacgguauacAagugu....................   1      1     Mm3
.....................................................................aagcaccacgguauaccGgugu....................   2      1     Mm3
.....................................................................aagcaccacgguauaccaguUu....................   2      1     Mm3
.....................................................................aagcaccacgAuauaccagugu....................   1      1     Mm3
.....................................................................aagcaccacgguauaccCgugu....................   2      1     Mm3
.....................................................................aagcaccacgguauaccagugu....................   300    0     Mm3
.....................................................................aagcaccacgguauacUagugu....................   2      1     Mm3
.....................................................................aagcaccacgguauaccUgugu....................   2      1     Mm3
.....................................................................aagcaccacgguauaccaguguA...................   1      1     Mm3
.....................................................................aagcaccacgguauaccaguguU...................   4      1     Mm3

..................................acuguuaucuccugguggauuug......................................................   14     0     Fm1
.....................................................................aagcaccacgguauaccagu......................   1      0     Fm1
.....................................................................aagcaccacgguauaccagug.....................   5      0     Fm1
.....................................................................aagcaccacgguauaccCgugu....................   6      1     Fm1
.....................................................................aagcaccacgguauaccagugu....................   2265   0     Fm1
.....................................................................aagcaccacgguGuaccagugu....................   2      1     Fm1
.....................................................................Uagcaccacgguauaccagugu....................   1      1     Fm1
.....................................................................aagcaccacgCuauaccagugu....................   4      1     Fm1
.....................................................................aagcaccUcgguauaccagugu....................   1      1     Fm1
.....................................................................aagcaccacgguauaccaguCu....................   1      1     Fm1
.....................................................................aagcaccacgAuauaccagugu....................   7      1     Fm1
.....................................................................aagAaccacgguauaccagugu....................   1      1     Fm1
.....................................................................aagcaccacgguauacGagugu....................   2      1     Fm1
.....................................................................aagcaccacgguauaccagugC....................   1      1     Fm1
.....................................................................aagcaccacgguauacUagugu....................   7      1     Fm1
.....................................................................aagcaccacgguauaccGgugu....................   5      1     Fm1
.....................................................................aagcaccacgguauaccagCgu....................   2      1     Fm1
.....................................................................aagcaNcacgguauaccagugu....................   1      1     Fm1
.....................................................................aagcaccacgguauaccagugA....................   2      1     Fm1
.....................................................................aagcaccacgguauaccagugG....................   250    1     Fm1
.....................................................................aagcaccacgguauacAagugu....................   11     1     Fm1
.....................................................................aagcaUcacgguauaccagugu....................   1      1     Fm1
.....................................................................aaUcaccacgguauaccagugu....................   3      1     Fm1
.....................................................................aagcaccacgguauaccUgugu....................   10     1     Fm1
.....................................................................Gagcaccacgguauaccagugu....................   1      1     Fm1
.....................................................................aagcaccacggCauaccagugu....................   1      1     Fm1
.....................................................................aagcacUacgguauaccagugu....................   2      1     Fm1
.....................................................................aagcaccacgguauUccagugu....................   1      1     Fm1
.....................................................................Nagcaccacgguauaccagugu....................   2      1     Fm1
.....................................................................aagcaccacgguauaccaguguA...................   3      1     Fm1
.....................................................................aagcaccacgguauaccaguguU...................   113    1     Fm1
.....................................................................aagcaccacgguauaccaguguUa..................   1      1     Fm1
......................................................................agcaccacgguauaccagugu....................   3      0     Fm1

..................................acuguuaucuccugguggauuug......................................................   12     0     Fm2
..................................acuguuaucuAcugguggauuug......................................................   1      1     Fm2
.....................................................................aagcaccacgguauaccagu......................   4      0     Fm2
.....................................................................aagcaccacgguauaccagug.....................   7      0     Fm2
.....................................................................aagcaccacgguauaccaguA.....................   3      1     Fm2
.....................................................................aagcaccacgguauaccGgugu....................   1      1     Fm2
.....................................................................aagcaccacgguauaccagugG....................   29     1     Fm2
.....................................................................aagcaccacgguauaccagugu....................   400    0     Fm2
.....................................................................aagcaccacgguauaccUgugu....................   1      1     Fm2
.....................................................................aagcaccacgguaCaccagugu....................   1      1     Fm2
.....................................................................aagAaccacgguauaccagugu....................   1      1     Fm2
.....................................................................aagcaccaUgguauaccagugu....................   1      1     Fm2
.....................................................................aUgcaccacgguauaccagugu....................   1      1     Fm2
.....................................................................aagcaccacgguauaccCgugu....................   3      1     Fm2
.....................................................................aagcaccacgguauacGagugu....................   1      1     Fm2
.....................................................................aagcaccacgguauacUagugu....................   2      1     Fm2
.....................................................................aagcaccacgCuauaccagugu....................   1      1     Fm2
.....................................................................aagcaccacgguauaccaguguU...................   64     1     Fm2
.....................................................................aagcaccacgguauaccaguguUa..................   13     1     Fm2

..................................acuguuaucuccugguggau.........................................................   1      0     Mm2
```

Star Mature

```
aacuugacaaugacuucaaucucaaggcacuugcacuguuaucuccugguggauuugucuaauauaccaaagcaccacgguauaccagugugaauugucugaauuccuacg
..................................acuguuaucuccugguggauuu.......................................................  1     0  Mm2
..................................Ucuguuaucuccugguggauuug......................................................  2     1  Mm2
..................................acuguuaucuccugguggauuug......................................................  34    0  Mm2
..................................acuguuaucuccugguggauuugu.....................................................  1     0  Mm2
.....................................................................aagcaccacgguauaccagu......................  13    0  Mm2
.....................................................................aagcaccacgguauaccagug.....................  20    0  Mm2
.....................................................................aagcaccacgguauaccagGg.....................  1     1  Mm2
.....................................................................aagcaccacgguauaccaguA.....................  1     1  Mm2
.....................................................................Uagcaccacgguauaccagugu....................  1     1  Mm2
.....................................................................aagcaccacgguauaccGgugu....................  8     1  Mm2
.....................................................................aagcaccacgguauaccagugu....................  2686  0  Mm2
.....................................................................aGgcaccacgguauaccagugu....................  2     1  Mm2
.....................................................................aagcaccacgUuauaccagugu....................  1     1  Mm2
.....................................................................aagcaccacgguauacUagugu....................  5     1  Mm2
.....................................................................aagcaccacgguauacGagugu....................  6     1  Mm2
.....................................................................aagcaccacgguauaccUgugu....................  11    1  Mm2
.....................................................................aagcaccacgAuauaccagugu....................  5     1  Mm2
.....................................................................aagcaccacgguauaccagugG....................  176   1  Mm2
.....................................................................aagcaccacgCuauaccagugu....................  5     1  Mm2
.....................................................................aagcaccacCguauaccagugu....................  1     1  Mm2
.....................................................................aaUcaccacgguauaccagugu....................  1     1  Mm2
.....................................................................aagcaccacgguauaccCgugu....................  9     1  Mm2
.....................................................................aagUaccacgguauaccagugu....................  1     1  Mm2
.....................................................................aagcaccacAguauaccagugu....................  1     1  Mm2
.....................................................................aagcaccacgguaCaccagugu....................  2     1  Mm2
.....................................................................Nagcaccacgguauaccagugu....................  1     1  Mm2
.....................................................................aagcaNcacgguauaccagugu....................  1     1  Mm2
.....................................................................aagcaccacgguauacAagugu....................  19    1  Mm2
.....................................................................aagcacAacgguauaccagugu....................  1     1  Mm2
.....................................................................aCgcaccacgguauaccagugu....................  1     1  Mm2
.....................................................................aagcaccacgguauaccagugC....................  3     1  Mm2
.....................................................................aagcaccacgguaGaccagugu....................  1     1  Mm2
.....................................................................aagcGccacgguauaccagugu....................  2     1  Mm2
.....................................................................aagcaccacgguauaccagugA....................  3     1  Mm2
.....................................................................aagcaccacgguauUccagugu....................  1     1  Mm2
.....................................................................aagcaccacgguauaccaguguU...................  35    1  Mm2
.....................................................................aagcaccacgguauaccaguguA...................  7     1  Mm2
......................................................................agcaccacgguauaccagugG....................  1     1  Mm2
......................................................................agcaccacgguauaccagugu....................  5     0  Mm2

..................................acuguuaucuccugguggauuug......................................................  13    0  Mm1
.....................................................................aagcaccacgguauaccagu......................  8     0  Mm1
.....................................................................aagcaccacgguauaccagug.....................  31    0  Mm1
.....................................................................aagcaccacgAuauaccagugu....................  2     1  Mm1
.....................................................................aagcaccGcgguauaccagugu....................  2     1  Mm1
.....................................................................aagcaccacgguauacGagugu....................  1     1  Mm1
.....................................................................aagcaccacgguauacAagugu....................  4     1  Mm1
.....................................................................aagcaccacgguauaccGgugu....................  1     1  Mm1
.....................................................................aagcaccacgguauacUagugu....................  1     1  Mm1
.....................................................................aagcaccacgguauaccUgugu....................  2     1  Mm1
.....................................................................aagcaccacgguauaccagugu....................  630   0  Mm1
.....................................................................Uagcaccacgguauaccagugu....................  1     1  Mm1
.....................................................................aagcaccacgguauaccagugG....................  9     1  Mm1
.....................................................................aagcaccacgguauaccagCgu....................  1     1  Mm1
.....................................................................aagcaccacgguauaccaguguU...................  11    1  Mm1
```
